# Observations upon Tetanus

**Published:** 1810-01

**Authors:** J. Howship

**Affiliations:** Surgeon


### *Observations upon Tetanus; by Mr.* J. Howship, *Surgeon.* *In Continuation.*

It has been already observed, that in certain points of character, the spasm of tetanus, and that of an epileptic attack, may be considered to bear some resemblance to each other. At the same time it has been pointed out, that in essential circumstances, apparent enough at the commencement, as well as upon the establishment of each of these diseases, they are very distinct in their nature. If this distinction is to be made out in the early progress of these diseases, it will be found to be still more evident and clear, in considering the modes of termination, which are peculiar to each of these complaints.

The epileptic disease, in its first attack, is most usually of very short duration; although, for the period of its continuance, the senses are abolished, the convulsions are generally less intense, than in the subsequent attacks of this complaint.

The recovery from the fit takes place readily, and the mental powers are at once fully re-established; very little, if any, soreness in the muscular parts of the body and limbs is felt; and, during the period of the interval, the various powers, relating both to the corporeal and mental constitution, seem to remain completely unimpaired.

The disease, however, continues to exist, and the fits return after a greater or a less distant period of time has elapsed. A particularly severe fit shall be longer than usual in duration; the convulsive actions of the limbs more violent and unmanageable than common; and, after the cessation of this attack, the patient will be observed to recover his mental powers more slowly than he formerly did. He opens his eyes, and looks about him with a sort of wild curiosity; very expressive of the truth, that he does not recollect either where or who he is, nor in what circumstances he is placed: thus it will, sometimes, be many hours before the memory is perfectly recovered.

Towards the close of the disease, the visible influence produced by the complaint upon the intellect, becomes daily more and more severe. The memory is more frequently suspended, until finally it is abolished; the judgment becomes weakened, and at last this also sinks altogether.

Very rarely it happens that a most violent fit of convulsion is suddenly followed by the fatal event; much more frefrequently the severity of the attack produces extravasation from some of the small vessels, either within the substance of the brain, or upon some part of its membranes. In the latter case, the convulsion gradually subsides into a state of coma, an apoplectic sleep from which the patient does not recover.

From the above it is sufficiently manifest, that although at the commencement of epilepsy the mental powers may be very perfect and clear, yet, for some time preceding the unfortunate termination of this disease, they are in almost every instance totally abolished.

The progress of tetanus, although a disease equally fatal with epilepsy, is yet unattended with that abolition or confused state of intellect, which is uniformly produced by those disorders consequent to a compressed state of the brain.

In tetanus the spasms, from being at first slight, become afterward more severe; and the attacks more permanent, as well as more universal, than they were before. The disease seems to influence progressively the whole of the nervous system; and unless a something, that has been supposed to be a radical strength, exists in the constitution, the complaint eventually proves fatal; for it latterly reaches the organs of respiration and circulation, inducing a state of spasm in the ventricles of the heart. The intellectual powers, however, constantly remain clear throughout; and up to the very moment of the fatal event, reason is still perfect.

The respective lines of distinction that exist between one disease and another, are of the highest importance in practice. Not that there is much probability of epilepsy being mistaken for tetanus; but the important fact is this, that upon a just estimation of the true nature of the symptoms, the treatment and management must depend.

Even the best conclusions, those drawn from practice, are sometimes deceptive. A case of epilepsy has, in some rare instances, when the disease has reached its latter stage, taken on, in some measure, the form and appearances of tetanus, the body and limbs having been variously distorted with a rigid spasm; and these cases of very rare occurrence seem to have introduced an opinion, that the two diseases are, in fact, the same; and that the various forms of tetanus are only so many different species of a particular epileptic paroxysm, the same mode of treatment being necessary and proper in both complaints.

The dangerous and even destructive tendency of such a conconclusion as the above in practice, must be sufficiently clear. How many instances are upon record, of the epileptic fit having brought on one or other variety of paralysis, from which the patient has never recovered? Such an event has never been observed to take place in an attack of tetanus. It may perhaps be objected, that tetanus cannot exhibit the same phenomena with epilepsy, from the wide difference in the length of period assigned to each complaint; although the two diseases may be still very nearly the same. It is unquestionably true, that tetanus always runs a rapid course, when compared with epilepsy; but notwithstanding this, seeing that the symptoms denoting a turgid state of the vessels within the head, or a compressed state of the brain, are so numerous and so well known, can it be supposed that such state can ever subsist in the living body, without in some shape being discerned by an attentive Practitioner, more particularly while the patient has the power of expressing whatever he feels of pain or inconvenience?

In the treatment of the epileptic disease, where the brain has manifestly sustained an injury, either from the formation of disease, or from the effusion of some portion of the circulating fluids within the cavity of the skull, the only useful principle of action suggests the immediate removal of a part of the fluid contents of the head; this first intention being assisted by the adoption of such measures, as may still further be conducive to the same end, by the producing a state of excitement in the absorbents of the brain.

What would such a practice be in a case of tetanus? Would it not be both useless and hurtful? There is some reason to believe, that in the treatment of tetanus, the most flattering prospeet of recovery might, in general, be afforded, by the early and free exhibition of opium, assisted by wine and bark. Would such a plan of treatment fail to destroy in any case, in which the brain had already suffered under the oppressive influence of the epileptic disease?

In a case of questionable character, where the epileptic paroxysm might be succeeded by the appearances and spasms peculiar to tetanus, it would certainly be proper to consider and weigh very carefully the prevailing circumstances of the disease; in order that an accurate estimate may be made, and the treatment conducted upon the best principles. In such acase, the plan of cure should be governed by the prevailing disposition of the malady; which which would certainly incline very manifestly either towards the epileptic character, or to that of tetanus.

Should the case have been originally an epilepsy, and the circumstances of the history such, as to leave no ground for presuming the existence of any organic derangement of structure about the brain, there would be, comparatively, little objection to the adoption of such a system of treatment, as might probably succeed in removing any complaint, approaching closely in its characters to the spasm of tetanus.

The cases that would, with probable safety, admit the adoption of a plan of cure that might succeed, where the spasm is considered to assume the aspect of tetanus, are mostly those in which the epileptic fit has usually given some description of warning of its approach; either by a sensation of giddiness, or peculiar affection of the head, or a creeping or numbness felt upon some part of the surface in the immediate neighbourhood of the head.

The most favorable case, however, for such treatment, would appear to be one, in which the fit has been preceded by that very curious sensation, the aura epileptica. A very singular affection, generally commencing in the extreme point of some one nerve, and slowly proceeding thence towards the head, generally depriving that particular nerve of its proper energy, and therefore producing a sense of numbness, or torpor, extending along its course, till the impression has reached the brain, when the fit immediately comes on; and after this the irritation is not again felt, until the next following attack of convulsion is about to take place.

There is something in the nature of such an impression as the above, that is, in particular instances, highly curious, and indeed very much involved in mystery. The epilepsy from it springing from the aura epileptica, fairly admits of being considered as a parallel case with tetanus, arising from a wound in an extremity, in which the occasional startings and uneasiness in the part wounded, forewarns the patient of what he is about to suffer. The consequent spasms by which the body is agonised in such a case, admits of probable explanation, upon the presumption that a remote irritation existing in a part of the body, the impression is conveyed to the sensorium, and the convulsions that follow, only prove the intimate dependence that subsists in every living body, between those structures that are themselves endowed with the power of motion, motion, and those others, in which is resident the active principle, by the agency of which motion is produced.

In confirmation of the idea, that an epileptic aura always proceeds from some derangement in the organisation of the part in which it is perceived to arise, it has very generally been detected springing from a situation, in which either wound or bruise has been received: nay, the conviction that it must arise from some material alteration in structure, has been so strong, that it has even urged the Surgeon to plunge his knife through sound integuments, and the thick mass of muscle posterior to the tibia; where, directed by the uniform sensations of the patient, he has found a small hard cartilaginous body, that lay in contact with, and pressed upon, one of the nerves of the leg; the removal of which tumor, by the division of the nerve, was the efficient means of curing the complaint.

It frequently, however, appears, that a close attention to diseases, is apt to unsettle opinions that have very long been received. The aura epileptica, not unfrequently, is found to arise in a part where no visible complaint can be traced, or ever existed. No tenderness to the touch, no indurations, nor change of colour; but even then it might be a probable conjecture, that some derangement in the condition had taken place, although confined to the more secret and minute organization, and therefore, not capable of being detected by the senses. But if these cases are of doubtful explanation, what shall be said to one in which the aura existing in the extremity of a limb, has, without the agency of any visible cause, suddenly shifted its seat, springing the very next day, with equal effect, from a distant and distinct point from that in which it was first perceived?—So curious a phenomenon can, perhaps, only be referred to some cause existing primarily in the very source or centre of the nervous system, some peculiar condition of the brain.

The similarity that may be traced between a case of epilepsy arising from a remote irritation, and a case of tetanus produced by a wound, very naturally suggested the probability, that a certain mode of treatment, that experience had proved to be useful in the one case, might also be adopted with advantage in the other.

The experiment was made, and the result was so far fortunate as to render the case deserving of notice.

In the following case was also ascertained, how far a moderate degree of pressure is capable of restraining the progress of the morbid influence of a diseased impression,

 when when passing upwards from the extremity of a nerve towards the brain.

---

*Case of Epilepsy, in which the Recurrence of the Paroxysm was prevented by the Use of Cold Affusion.*

Samuel Montgomery, aged 23 years, private in the 9th Regiment of Light Dragoons, when about 19, was first attacked by the disease. He had taken very violent exercise, and was in a profuse perspiration when he sat down to rest himself. When cooled, he felt himself becoming heart-sick and heavy. He went, however, and got into bed, slept very well, and the next morning felt, for the first time, a very singular sensation in the right thumb, "starting, jumping and leaping," the impression from which in a very short time reached the brain, when he became insensible.

In this state he remained some time, and when he came to himself, he found his body and limbs altogether very stiff and sore, as if they had been bruised.

During that day he had as many as nine fits: but afterward the paroxysms became less frequent. For the removal of his complaint, he about this time took some medicines, that produced very free discharges by perspiration. The fits soon after left him, and he had none for three months. The next fit seized him while on the road to a market; this also was preceded by the sensation which he had formerly observed in his right thumb. His companions carried him into a house, and the next morning he applied himself to a priest that lived in the village. The priest forbid his eating meat, and desired him never to enter a church.

For three years after this he was free from the complaint, when he was again attacked while sitting drinking in a canteen, which custom he believed to have been the means of bringing back the disorder. It then commenced as before in the thumb of the right hand, passing gradually upwards, and producing convulsive startings, first in the muscles of the thumb, and then in progression; bringing on precisely the same action in those upon the fore-arm, arm, and shoulder. After this time it returned on the average every three weeks or a month.

While passing the island of Madeira, on his passage to South America, this man was attacked by his old complaint, and had several severe fits. He had generally had his grog regularly since he had been at sea, but on the day he was attacked he had taken none. He had two fits the same afternoon. He had taken the precaution once suggested gested to him, of having a tight ligature made round the arm on the affected side, this he had produced by a twisted pocket handkerchief, and he said; he believed that it did in some degree retard the progress of the impression upwards from the thumb, but he never was able to prevent its reaching the head at last, so never escaped a fit by that degree of ligature which he had it in his power to make.

In Porta Praya, upon the island of St. Tiego, one of the Cape de Verds, he felt the same sensation that formerly had been confined to his right thumb, now arising in his left, although only the day before he had had a fit, when the aura commenced from its old point.

Directions had been given that the moment the attendants were apprized of an approaching fit, he should be brought quickly as possible upon deck, to the forecastle of the ship, for the purpose of ascertaining the effect of the cold affusion. He said to his companions that he now felt exactly the same sort of starting and jumping in the thumb of the left hand, that before used to take place on the right side, and that of course he knew he should very quickly have a fit. Upon this intimation he was immediately taken up, his clothes were stript off by some, while others assisted by collecting buckets of salt water from along side, and in this way he had several pails of water dashed over his head and body, before the impression from the nerve in the thumb had reached the head. The promptness and force of the application was such, that it several times deprived him almost of the power of recovering his breath. Between twelve and eighteen full buckets were thrown over him, and the effect was decidedly satisfactory. The impression not only was prevented from reaching the brain, but it was felt no more, and although he had for more than a month before, had a fit almost daily, the complaint did not return upon him for the time that he was upon the passage southward, in the tropical latitudes, nor for the rest of the voyage had he any attack.

His expression, with regard to the effect of the experiment was, that the cold water "startled him" very much; he also observed that he was very much "surprised" by it.

*Mill Street, Hanover Square,*
*December* 15, 1809.

( To be continued. )

